# Altered human gut virome in patients undergoing antibiotics therapy for *Helicobacter pylori*

**DOI:** 10.1038/s41467-023-37975-y

**Published:** 2023-04-17

**Authors:** Lingling Wang, Haobin Yao, Daniel C. Morgan, Kam Shing Lau, Suet Yi Leung, Joshua W. K. Ho, Wai K. Leung

**Affiliations:** 1 Department of Medicine, School of Clinical Medicine, The University of Hong Kong, Hong Kong, China; 2 School of Biomedical Science, The University of Hong Kong, Hong Kong, China; 3 Laboratory of Data Discovery for Health, Hong Kong Science Park, Hong Kong, China; 4 Centre for PanorOmic Sciences (CPOS), The University of Hong Kong, Pokfulam, Hong Kong, China; 5 Department of Pathology, The University of Hong Kong, Queen Mary Hospital, Pokfulam, Hong Kong, China; 6 The Jockey Club Centre for Clinical Innovation and Discovery, LKS Faculty of Medicine, The University of Hong Kong, Pokfulam, Hong Kong, China

**Keywords:** Stomach diseases, Microbial ecology, Gastrointestinal diseases

## Abstract

Transient gut microbiota alterations have been reported after antibiotic therapy for *Helicobacter pylori*. However, alteration in the gut virome after *H. pylori* eradication remains uncertain. Here, we apply metagenomic sequencing to fecal samples of 44 *H. pylori*-infected patients at baseline, 6-week (*N* = 44), and 6-month (*N* = 33) after treatment. Following *H. pylori* eradication, we discover contraction of the gut virome diversity, separation of virome community with increased community difference, and shifting towards a higher proportion of core virus. While the gut microbiota is altered at 6-week and restored at 6-month, the virome community shows contraction till 6-month after the treatment with enhanced phage-bacteria interactions at 6-week. Multiple courses of antibiotic treatments further lead to lower virus community diversity when compared with treatment naive patients. Our results demonstrate that *H. pylori* eradication therapies not only result in transient alteration in gut microbiota but also significantly alter the previously less known gut virome community.

## Abstract (web summary)

Here, Wang et al. use metagenomic sequencing to explore the impact of antibiotic treatment for *Helicobacter pylori* on the gut virome community in infected patients, showing that recurrent treatment leads to a lower virus community diversity and altered virus-bacteria interactions, compared with treatment naive patients.

## Introduction

It was estimated that 4.4 million individuals in the world were infected with *Helicobacter pylori*, particularly in Asia where up to 54.7% of population were infected^1^. *H. pylori* could cause a broad range of gastrointestinal diseases including peptic ulcer, gastric cancer, and mucosa-associated lymphoid tissue lymphoma (MALT)^2^. While eradication of *H. pylori* by antibiotics could lower the risk of these gastrointestinal diseases, antibiotic treatment can also accidentally reshape the gut microbiota^3,4^ and impact on host health^5–7^. Increasing evidence suggests that while gut bacteria play a prominent role in disease development and progression^8^, factors including diet, lifestyle, host genetics, age, and geographic factors could also contribute to gut bacterial profiling^9^. Thus far, microbiota studies have demonstrated significantly altered gut microbiota, their associated metabolic functions and interactions with host, after antibiotics treatment for *H. pylori*^10,11^.

While the human gut microbiome is a complex environment composed of bacteria, viruses, archaea, and fungi^12^, most researchers have focused on the changes in gut bacteria after *H. pylori*-eradication. Nonetheless, the changes in gut virome and its correlation with the host after treatment for *H. pylori* remains unclear. The healthy gut virome comprises bacteriophages (which infect bacteria), eukaryotic viruses and archaea viruses, which is relatively stable and highly individual-specific with a predominance of *Caudoviricetes* class (mainly bacteriophages)^13–16^. Bacteria serve as the natural hosts for bacteriophages, and bacteria-phage coevolution is regarded as a crucial driver of the ecological and evolutionary process of the bacteria community^17^. Antibiotics used in the eradication therapy may promote the enrichment of some resistant strains, like *Escherichia coli*, or removal of targeted bacteria species^18^. Given the effects of antibiotics on microbiota, the impact on bacteriophages is less predictable as the alteration is the result of both phage-bacteria interaction and phage-antibiotic synergy.

Recent studies have suggested the potential associations between gut virome and human diseases including type 2 diabetes, irritable bowel syndrome and non-alcoholic fatty liver disease^19–21^, but the functions or mechanisms underlying the altered viral species were not yet fully understood. Possible mechanisms including the interactions between gut virome and bacteria with reshaping of gut microbiota through lytic or lysogenic life cycles and improving bacteria function through horizontal gene transfer (HGT), which may contribute to human disease pathogenesis^22^. Understanding the alteration in the gut virome associated with *H. pylori* eradication may also help to optimize current *H. pylori* eradication regimens or alleviating the adverse effects of antibiotic treatment through manipulating the microbiome, like fecal virome transplantation (FVT) or phage therapy to reduce the antibiotic resistance.

With the availability of next-generation sequencing, we can further explore the previously non-culturable and unclassified microbes. Several cross-sectional and longitudinal studies have revealed disease-specific gut virome dynamics and various virus-bacteria dynamics^21,23–25^. The NCBI viral RefSeq, the Gut Virome Database (GVD), Integrated Microbial Genomes database (IMG/VR), and the Gut Phage Database (GPD, only phage genome) are frequently used viral sequence databases^13,26,27^. Through these viral reference databases, we can explore the gut virome dynamics after receiving *H. pylori* eradication.

In this work, we address the changes in gut virome after *H. pylori* eradication. We have collected serial stool samples from patients who have received *H. pylori* eradication therapy and conducted metagenomic sequencing to determine the longitudinal changes in gut virome, microbiome, and virus-bacteria interactions, before and after eradication treatment.

## Results

### Alteration of viruses at different prevalence levels

Overall, we identified 3581 different viral species from 18,005 qualified contigs, including 12 core viruses (viral species found in over 50% of the samples), 66 common viruses (found in 20–50% of the samples), and 3503 unique viruses (found in less than 20% of the samples), suggesting an individual-specific gut virome pattern. All of the core viruses were bacteriophages, including *Faecalibacterium* phages (*Brigitvirus brigit, Eponavirus epona*, *Lagaffevirus lagaffe*, *Mushuvirus mushu*, and *Toutatisvirus toutatis*), *Bacteroides* phage (*CrAss-like virus* sp.), *Clostridium* phage (*Clostridium phage c-st*), *Flavobacterium* phage (*Immutovirus immuto*), *Priestia* phage (*Donellivirus gee)*, *Pseudomonas* phage (*Knuthellervirus PMBT14*), *Salmonella* phage (*Salmonella phage 7-11*), and *Vibrio* phage (*Vibrio phage henriette 12B8*), whose host was abundant intestinal bacteria (Supplementary Fig. 1). Among which, *crAssphage* was reported to be highly abundant in human fecal metagenomics^28^.

**Fig. 1:**
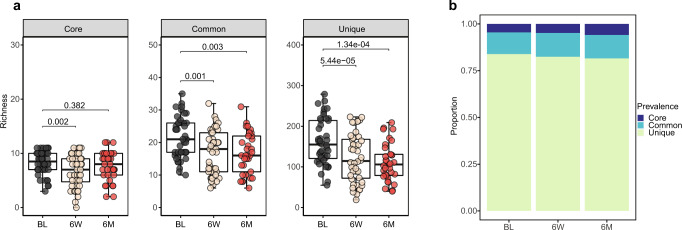
Alteration of core, common, and unique viruses after *H. pylori* eradication therapy. **a** Boxplots show the comparison of richness of viral species at each prevalence level (core, common, and unique) among the three groups (two-sided Wilcoxon tests and adjusted by Benjamini–Hochberg (BH) method for multiple comparisons; Baseline, *N* = 44; 6-week, *N* = 44; 6-month, *N* = 33). **b** Stacked plots show the proportion of core, common, and unique viral species at baseline (BL, *N* = 44), 6-week (6 W, *N* = 44), and 6-month (6 M, *N* = 33). The boxes denote the lower 25% quantile, upper 75% quantile, and center line the median, with whiskers extending to a limit of ±1.5 interquartile ranges (IQRs).

The core virus richness diminished 6-week after *H. pylori* eradication therapy (FDR = 0.002) and gradually restored at 6-month (FDR = 0.382); while common and unique viruses showed a decline both at 6-week (FDR = 0.001 and FDR = 5.44e−05) and 6-month (FDR = 0.003 and FDR = 1.34e−04) after eradication (Fig. 1a). The Sankey diagram indicated the transition from core to common/unique viruses in most of the samples after eradication therapy, consistent with the diminishing of virus richness at different prevalence levels (Supplementary Fig. 2a). We next evaluated the alteration in viral community composition after eradication (Fig. 1b), which indicated that the proportion of core viruses increased (FDR = 0.001), but unique viruses (FDR = 0.006) decreased at 6-month (Supplementary Fig. 2b). These results suggested that the core viruses were more resilient to external challenges like antibiotic exposure compared to common and unique viruses.

### Altered virome diversity and composition after eradication therapy

**Fig. 2:**
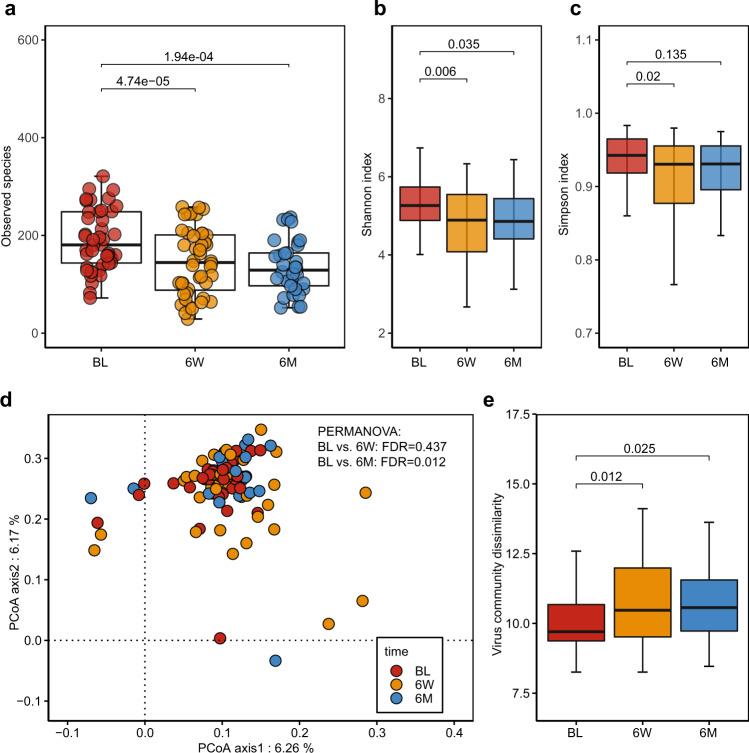
Biodiversity alteration of the gut virome community after *H. pylori* eradication therapy. **a**–**c** Comparisons of alpha diversity indices in virus richness (**a**), Shannon index (**b**), and Simpson index (**c**) among the three groups (BL: *N* = 44; 6 W: *N* = 44; 6 M: *N* = 33; *N*, the number of patients in each group). Two-sided Wilcoxon tests followed by BH correction were applied. **d** Principal Coordinate Analysis (PCoA) of the gut virome community among the three groups (BL: *N* = 44; 6 W: *N* = 44; 6 M: *N* = 33). Assessed via PERMANOVA test followed by *p*-value correction (BH method). **e** Comparison of the virome community dissimilarity distance at virus species level between baseline, 6-week and 6-month (BL: *N* = 44; 6 W: *N* = 44; 6 M: *N* = 33). The boxes denote the lower 25% quantile, upper 75% quantile, and center line the median, with whiskers extending to a limit of ±1.5 interquartile ranges (IQRs). All the tests were two-sided with BH method for multiple comparisons correction.

The gut virome diversity and community were significantly altered after eradication therapy. The alpha diversity of the gut virome significantly declined after *H. pylori* treatment. Compared to the baseline, the total virus richness (Fig. 2a) significantly decreased at 6-week (FDR = 4.74e−05) and 6-month (FDR = 1.94e−04). Both the Shannon index (Fig. 2b) and Simpson index (Fig. 2c) significantly decreased at 6-week (FDR = 0.006 and FDR = 0.02) and failed to return to baseline level 6-month after eradication therapy (FDR = 0.035 and FDR = 0.135). Using principal coordinates analysis (PCoA), we evaluated the beta diversity of the gut virome communities before and after treatment. We found that the baseline virome community was distinct from that at 6-month (PERMANOVA, FDR = 0.012) (Fig. 2d). Further comparison of the average community dissimilarity (Fig. 2e) confirmed that the viral community difference was significantly higher at 6-week (FDR = 0.012) and 6-month (FDR = 0.025) when compared to baseline level, indicating the difference in virome community under the pressure of antibiotic-based eradication.

We next performed differential analysis to investigate the alteration in gut virome composition after eradication therapy at various taxonomy levels. At the class level, the *Caudoviricetes* class (tailed bacteriophages) was predominant, accounting for over 90% of the viral composition (Supplementary Fig. 3a), while most of them were not assigned at order, family, or genus level (Supplementary Fig. 3b–d). Compared with baseline, the abundance of the *Caudoviricetes* class showed no significant alteration after eradication therapy (Supplementary Data 3). Further analysis of the alpha diversity indices (Supplementary Fig. 4a) of *Caudoviricetes* class showed that richness, Shannon index, and Simpson index were all significantly decreased at 6-week (FDR = 2.28e−05, FDR = 0.004, and FDR = 0.012, respectively) and 6-month (FDR = 1.65e−04, FDR = 0.05, and FDR = 0.18, respectively). The beta diversity (Supplementary Fig. 4b) of *Caudoviricetes* class was separated from baseline at 6-month (PERMANOVA, FDR = 0.024) with increased community structure dissimilarity at both 6-week (FDR = 0.006) and 6-month (FDR = 0.023).

**Fig. 3:**
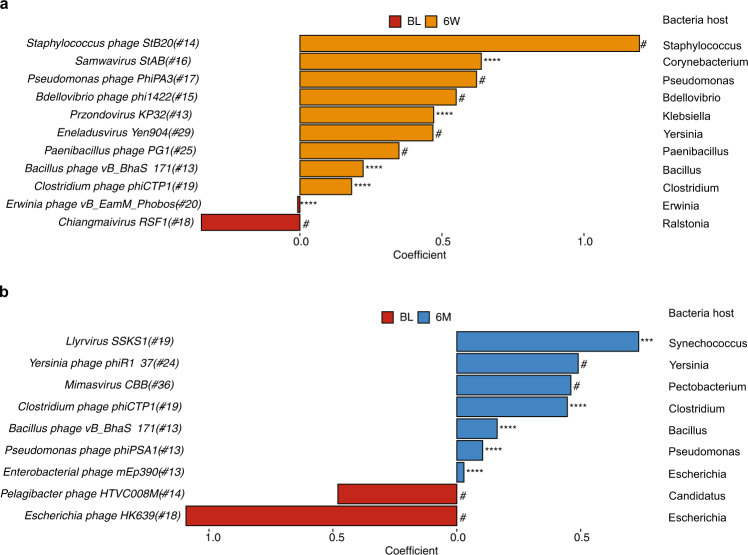
Significantly altered viral taxa after *H. pylori* eradication therapy. **a**, **b** Significantly altered viral taxa at (**a**) 6-week and (**b**) 6-month compared to baseline using MaAsLin2 (adjusted for age, gender, treatment regimens, drug durations, and the number of raw reads; baseline was set as reference group). Coefficient was obtained from MaAslin2 output and only those significantly altered taxa were presented (original *p* < 0.05 with qval < 0.15). Positive coefficient value represented viral taxa enriched at 6-week or 6-month, while negative coefficient value represented viral taxa enriched at baseline. The number following the species name suggested the prevalence of the bacteriophage. (#qval < 0.15, *pqval < 0.05, **qval < 0.01, ***qval < 0.001, ****qval < 0.0001; all original pval < 0.05).

We applied MaAslin2 to identify the viral taxa that were closely associated with *H. pylori* eradication. At 6-week, we observed 11 bacteriophages that were significantly altered (Fig. 3a and MaAslin2 output in Supplementary Data 3) and of which 9 were enriched, including *Bacillus* phage (*Bacillus phage vB_BhaS-171*), *Bdellovibrio* phage (*Bdellovibrio phage phi1422*), *Clostridium* phage (*Clostridium phage phiCTP1*), *Corynebacterium* (*Samwavirus StAB*), *Klebsiella* phage (*Przondovirus KP32*), *Paenibacillus* phage (*Paenibacillus phage pG1*), *Pseudomonas* phage (*Pseudomonas phage PhiPA3*), *Staphylococcus* phage (*Staphylococcus phage StB20*), and *Yersinia* phage (*Eneladusvirus Yen904*). The abundance of *Erwinia* phage (*Erwinia phage vB*_*EamM*_*Phobos*) and *Chiangmaivirus RSF1* diminished at 6-week. At 6-month (Fig. 3b and MaAslin2 output in Supplementary Data 3), there were 9 bacteriophages that were significantly altered, including *Bacillus phage vB_BhaS-171* and *Clostridium phage phiCTP1*. Other bacteriophages were also enriched at 6-month, including *Escherichia* phage (*Enterobacterial phage mEp390*), *Pseudomonas* phage (*Pseudomonas phage phiPSA1*), and *Yersinia* phage (*Yersinia phage phiR1-37*). Another *Escherichia* phage (*Escherichia phage HK639*) diminished at 6-month. Most of these altered bacteriophages after eradication were unique viruses with relatively low prevalence, suggesting that the low prevalence viral species were more likely to be affected by antibiotics, thus leading to the reconstruction of the gut virome community.

Together, these findings showed that *H. pylori* eradication therapy could result in depletion of gut virome richness, increase in virome community dissimilarity, and reshape of the virome composition.

### Different eradication therapies on gut virome alteration

We further explore the associations between different *H. pylori* eradication therapies and changes in gut virome. Firstly, we analyzed longitudinal alterations of the gut virome in patients with primary treatment (treatment naive) and retreatment (who have failed previous regimens) for *H. pylori*. The virus richness significantly decreased at 6-week and 6-month in both primary (Supplementary Fig. 5a) and retreatment groups (Supplementary Fig. 5b) (all FDR < 0.05). However, the beta diversity showed no significant separation in both groups (PERMAVONA, FDR > 0.05; Supplementary Fig. 5c, d).

Next, we compared the difference between the two treatment groups. Comparative analysis between the primary and retreatment groups showed that the retreatment group had lower virus richness at baseline, 6-week, and 6-month (all *p* < 0.05; Supplementary Fig. 6a–c), when compared to the primary treatment group. However, no significant separation was found between the two groups in beta diversity (PERMANOVA, all *p* > 0.05; Supplementary Fig. 6d–f). Although some bacteria and bacteriophage features were detected at baseline between those successfully eradicated and failed subjects (Supplementary Data 3), the functions of these were yet to be determined. Taken together, these results indicated that multiple eradication therapies could have a more profound effect on gut virome.

### Asynchronous recovery of virus and bacteria after eradication therapy

**Fig. 4:**
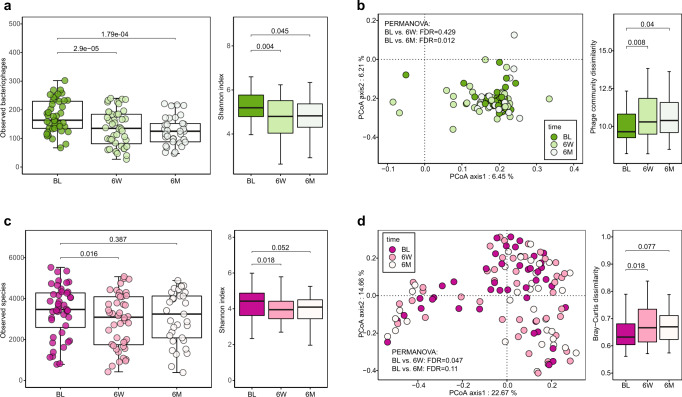
Biodiversity alteration of bacteriophages and bacteria after *H. pylori* eradication therapy. **a** Significantly altered bacteriophage alpha diversity in phage richness and Shannon index after eradication therapy (Wilcoxon tests followed by BH correction) between three groups (BL: *N* = 44; 6 W: *N* = 44; 6 M: *N* = 33; *N*, the number of patients included in each group). **b** Difference in bacteriophage community (beta-diversity) presented in PCoA plot based on phage community dissimilarity distance at the phage species level (PERMANOVA tests followed by BH correction) between three groups (BL: *N* = 44; 6 W: *N* = 44; 6 M: *N* = 33). Comparisons of distance of bacteriophages after eradication therapy. **c** Significantly altered bacteria alpha diversity in bacteria richness and Shannon index after the treatment (two-sided Wilcoxon tests followed by BH correction for multiple comparisons) between three groups (BL: *N* = 44; 6 W: *N* = 44; 6 M: *N* = 33). **d** PCoA plot shows the alteration of the bacteria community after the eradication therapy based on the bacterial community dissimilarity distance at the bacteria species level (PERMANOVA tests followed by BH correction) between three groups (BL: *N* = 44; 6 W: *N* = 44; 6 M: *N* = 33). Comparisons of dissimilarity distance of bacteria community after the eradication therapy. The boxes denote the lower 25% quantile, upper 75% quantile, and center line the median, with whiskers extending to a limit of ±1.5 interquartile ranges (IQRs).

Previous studies showed strong and significant correlations between bacteriophages and bacteria in various study populations^19,21,29^. We characterized all viral species according to their theoretical host. Since the proportion of bacteriophages dominated all other viruses (Supplementary Fig. 7), we first performed diversity analysis on bacteriophages and their associated microbiota. As for the bacteriophage, the phage richness and Shannon index decreased significantly at 6-week when compared to baseline (FDR = 2.9e−05 and FDR = 0.004) and failed to restore at 6-month (FDR = 1.79e−04 and FDR = 0.045) (Fig. 4a). PCoA analysis showed that the community structure of bacteriophage was significantly separated from baseline at 6-month (PERMANOVA, FDR = 0.012) with increased community dissimilarity (FDR = 0.04) (Fig. 4b). However, the composition of bacteriophages showed no significant alterations (Supplementary Data 3).

On the other hand, the diversity of gut bacteria was significantly altered but with a different trend. The bacteria richness and Shannon index decreased at 6-week (FDR = 0.016 and FDR = 0.018) but were restored to baseline level at 6-month (FDR = 0.387 and FDR = 0.052) (Fig. 4c). The beta diversity of bacteria was separated from baseline at 6-week (PERMANOVA, FDR = 0.047) which restored at 6-month (PERMANOVA, FDR = 0.11); and the Bray-Curtis dissimilarity of microbiota community displayed higher community difference at 6-week (FDR = 0.018), as compared to baseline and restored to baseline level at 6-month (FDR = 0.077) (Fig. 4d). The difference between the changes in bacteriophages and bacterial diversity indicated that the gut microbiota community was more resilient and easier to recover than the gut virome community after antibiotic-based *H. pylori* eradication treatment.

**Fig. 5:**
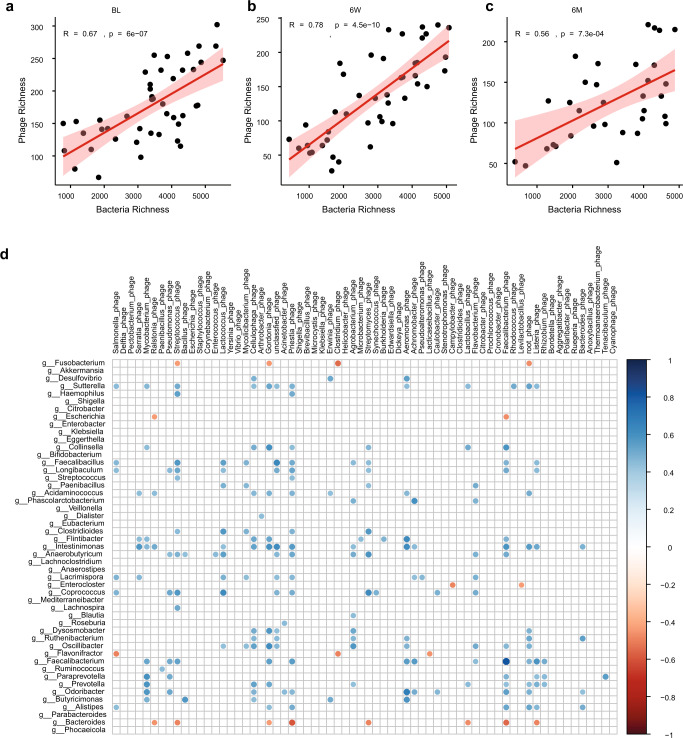
Alteration of virus-bacteria interactions after *H. pylori* eradication therapy. **a**–**c** Pearson correlations of alpha diversity indices (richness) between bacteriophages and bacteria at (**a**) baseline, (**b**) 6-week, and (**c**) 6-month. **d** Spearman correlations of phage-bacteria taxonomy 6-week after the eradication therapy. Rows indicated bacteria genera (*n* = 49). Columns indicated bacteriophages (*n* = 60, bacteriophages that infected the same bacteria genus were summed up. For example, *Escherichia phage D6*, *Escherichia phage VEc8*, and other viral species that infect *Escherichia* genus (bacteria) were summed up as *Escherichia phages*). Only significant correlations (adjusted *p* < 0.05) were displayed, detailed *p*-values were included in Supplementary Data 3. The color scale indicated the correlation strength. All the tests were two-sided with BH method for multi comparisons correction.

We further investigate the correlations between bacteriophage and bacteria on both alpha diversity index and taxonomy level. Bacteria and bacteriophage were all positively correlated at baseline (Pearson, *R* = 0.67, *p* = 6e−07; Fig. 5a), 6-week (Pearson, *R* = 0.78, *p* = 4.5e−10; Fig. 5b) and 6-month (Pearson, *R* = 0.56, *p* = 7.3e−04; Fig. 5c). We also evaluated the correlations between the bacteriophages and the bacteria genera. Overall, we observed a higher number of significant correlations (positive and negative) between phages and bacteria at 6-week (*n* = 213; Fig. 5d) than baseline (*n* = 13; Supplementary Fig. 8a) and 6-month (*n* = 4; Supplementary Fig. 8b). At 6-week, most of the significant correlations (91.07%, 194/213) were positive. While most negative correlations occurred between various bacteriophages and some bacteria genera, including *Bacteroides, Flavonifractor*, and *Fusobacterium* (Spearman, ρ range from −0.596 to −0.435; Fig. 5d). Most positive correlations occurred between bacteria genera (*Intestinimonas*, *Faecalibacterium, Sutterella*, *Anaerobutyricum*, *Odoribacter*, *Coprococcus*, *Faecalibacillus*, and *Oscillubacter*) and bacteriophages like *Faecalibacterium phage*, *Priestia phage*, *Lactococcus phage*, *Cellulophage phage*, *Streptococcus phage*, *crAssphage*, *Mycobacterium phage*. The abundance of *H. pylori* prophage (*Helicobacter phage phiHP33*) was negatively correlated with the *Helicobacter* genus (Supplementary Fig. 9; *R* = −0.24, *p* = 0.008). The correlation analysis indicated a transient but stronger association between phages and bacteria after antibiotic treatment at 6-week, which restored at 6-month, further confirmed the asynchronous restoration of phage and bacteria after treatment.

In addition to the previously reported positive bacteriophage-bacteria correlations after the antibiotic treatment^30^, the strong and positive correlations between other virus (non-bacteriophages) and bacteria alpha diversity indices (Supplementary Fig. 10) revealed undiscovered cross-kingdom associations among other viruses and bacteria. Similar to the alteration of phage-bacteria correlations after eradication therapy, the significance of the correlation between other viruses and bacteria tended to increase at 6-week (Pearson, *R* = 0.65, *p* = 1.8e−06; Supplementary Fig. 10b) and was restored at 6-month (Pearson, *R* = 0.0.58, *p* = 3.6e−04; Supplementary Fig. 10c), as compared to baseline (Pearson, *R* = 0.63, *p* = 5.4e−06; Supplementary Fig. 10a).

### Correlations between antibiotic resistance genes and bacteriophages

We also evaluated the interactions between antibiotic resistance genes (ARGs) and bacteriophages in view of the previous demonstration of significant correlation of gut ARGs with *H. pylori* eradication^31^. Consistent with previous study, various antibiotics treatments led to the enrichment of corresponding ARGs, including multidrug resistance genes (MDR) (*acrB*, *baeR*, *marA*, and *mdtP*), macrolide-lincosamide-streptogramin (MLS) (*Escherichia coli emrE* and *mphA*), fluoroquinolone (*Escherichia coli gyrA conferring resistance to fluoroquinolone* and *QnrS1*) and other ARGs. We found that *Rosemountvirus ZCSE2* was connected to a variety of ARGs glycopeptide resistance genes (*vanD*, *vanHD*, *vanXD*) at 6-week and *Cyprinid herpesvirus 2* was connected to various ARGs at 6-month (Supplementary Fig. 11a, b). We next summarized all the bacteriophages that share the same bacteria host at the genus level, the result showed that *Faecalibacterium* phage and *Aurantimonas phage* was positively correlated to a variety of ARGs (Supplementary Fig. 11c, d) at 6-week or 6-month. However, none of these single bacteriophage or summarized phages showed significant alteration after antibiotic treatment. We further established the network between bacteria genus and ARGs, and found enriched MDR genes (*acrB*, *baeR*, *marAm mdtP*) to be positively correlated with enriched *Shigella* and *Klebsiella* genera at 6-week (Supplementary Fig. 11e, f). These results suggest that the alteration of gut ARGs during the treatment was more likely to be associated with the bacterial host rather than bacteriophages.

## Discussion

To our knowledge, this is the first study to examine the longitudinal changes in gut virome in patients after *H. pylori* eradication therapy by using the whole-genome shotgun sequencing approach. Our results showed that there was a decrease in virus community diversity, an increase of gut virome difference, and reshaping of the virome community composition after antibiotics treatment for *H. pylori* eradication. Furthermore, different treatment regimens led to similar changes in gut virome, but repeated courses of *H. pylori* eradication therapies (in retreatment) could have a more enduring and stronger impact. While the gut microbiota was temporarily altered and was restored to normal at 6-month, the changes in diversity and structure of bacteriophages imposed by eradication therapy were more long lasting. Correlation analysis indicated that the antibiotic exposure promoted the coevolution process of bacteriophage and bacteria, with stronger positive or negative correlations at both diversity and taxa levels. The antibiotic treatment induced ARG enrichment was also more likely to be associated with changes in bacteria rather than bacteriophages.

In contrast to the high temporal intra-individual diversity in infants caused by vigorous colonization process^32^, healthy adult gut virome was relatively stable^13^. A previous study reported that 80% of viruses persisted in a healthy adult over a period of 2.5 years^33^. Cross-sectional studies have reported disease-specific gut virome alteration in non-alcoholic fatty liver disease (NAFLD)^21^, inflammatory bowel disease (IBD)^20^ including ulcerative colitis^23^, and type 1 or type 2 diabetes^19,34^ and colorectal cancer^35^. These studies indicate that the gut virome can be affected in various diseases, however, other environmental factors like medication (antibiotics or immunosuppressants), diet, geographical difference can also influence the bacterial and viral community of the gut^36,37^. Previous observations suggested prophage diversity was induced after antibiotic treatment in the gut of swine^38^, which was characterized by an expansion of phages after inhibition of bacteria by antibiotics^39^. Contrary to these findings, a study in autism spectrum disorder (ASD) patients who received microbiota transfer therapy consisting of 2-week antibiotic treatment showed that the gut phage diversity decreased after vancomycin treatment and recovered later in several subjects^40^. Similarly, we showed in this study that antibiotics used for *H. pylori* eradication could cause a decrease of virome (phage) diversity and increase in virome community dissimilarity, suggesting the recovery of gut intestinal inflammation or immune response and a healthier gut virome contributed by *H. pylori* eradication^41^.

Previous findings showed that various eradication therapies can lead to different and transient perturbation of the gut microbiota^4^, we observed both short- and medium-term gut virome alterations after various eradication regimens. One study reported that the phages may inhibit the growth of targeted bacteria and promote the enrichment of normal intestinal microenvironment and commensal^42^. Besides, majority of the bacteriophages identified in the human gut were temperate phages, which showed relatively lower mutation and evolution rates than the virulent bacteriophages, thereby leading to the different recovery paces of viral and bacterial communities^33^. Another possible reason for the different recovery paces may be caused by the spatially restructured bacterial community or microenvironment which contributes to the bacteria escape from antibiotic and phage attack^43^. Moreover, our results showed lower virome diversity in the retreatment group (who had failed prior eradication therapy) as compared to the primary treatment group up to 6-month after eradication therapy, further supporting the findings that the gut virome may be irreversibly altered by the eradication therapy and multiple courses of eradication therapies could have more long-lasting impacts on the virome community. Further large-scale studies involving various regimens will be needed to validate the impact of the different eradication regimens on the gut virome.

We observed transiently enhanced phage-bacteria correlations at 6-week at both diversity and taxonomy level, with an increased number of positive or negative bacteriophage-bacteria correlations, which may result from the complex phage-bacteria coevolution dynamic under antibiotic induced stress^44,45^. Moreover, we observed that antibiotic treatment could impact some of the gut bacteria^19^ and thus indirectly alter the abundance and prevalence of phages. For example, the abundance of *Bacillus*, *Bdellovibrio*, *Corynebacterium*, *Klebsiella*, and *Synechococcus* genera were enriched after the eradication therapy (Supplementary Data 3), corresponding to the enrichment of *Bacillus* phage (*Bacillus phage vB_BhaS-171*), *Bdellovibrio* phage (*Bdellovibrio phage phi1422*), *Corynebacterium* phage (*Samwavirus StAB*), *Klebs*iella phage (*Przondovirus KP32*), and *Synechococcus* phage (*Llyrvirus SSKS1*). *Bdellovibrio phage phi1422* was reported to be discovered in the blood of more than 50% of healthy individuals in a recent study^46^. As bacteria hosts can carry multiple phages at the same time, the alteration in a single bacteriophage may not follow the alteration of the bacteria host. For example, the abundance of *Enterobacterial phage mEp390* (*Escherichia* phage) enriched and *Escherichia phage HK639* diminished after eradication, while the abundance of *Escherichia* genus enriched after eradication. Yet, the functions of these bacteriophages would need to be validated by further studies. On the other hand, the enhanced bacteriophage-bacteria interactions and the abundant tailed bacteriophages from the class *Caudoviricetes* suggested that there were higher chances of phage-mediated horizontal gene transfer (HGT) in human gut^47^, such as transferring *phiHP33* orthologous genes to *H. pylori* strains, which was associated with *H. pylori* virulence markers cytotoxin-associated gene A (CagA) and vacuolating cytotoxin A (VacA)^48^.

In addition to the ability to infect the pathogens in the human gut, the bacteriophages or phage-derived particles (referred to as mobile genetic elements) are also capable of carrying and transferring antibiotic resistance genes into bacteria horizontally^49^ and promote the emergence of antibiotic-resistance intestinal flora^50,51^. However, our study showed that the enrichment of ARGs were more closely related to the bacteria host rather than bacteriophage. A previous study showed that none of the prophages identified carry known antimicrobial resistance genes, suggesting that ARGs are rarely encoded in phages and phages may not be responsible for ARG transfer^52,53^. The prevalence of *H. pylori* prophages is estimated to be ~21%^54^. Previous studies have identified the presence of *phiHP33* phage sequences and other prophages (*Schmidvirus KHP30*, *Schmidvirus KHP40*, and *Schmidvirus sv1961P*) in *H. pylori* strains^48,55^. Although no significant alteration was observed in these *H. pylori* phages, the *phiHP33* prophage showed significant correlation with the abundance of *Helicobacter* genus. Considering the presence of prophages in *H. pylori*, studies targeting the prophage genes related to the lysis cassette are useful for phage therapy^56^. In addition to the phage itself, the phage-encoded lysins and related bacteriolytic enzymes are also considered as potential alternatives to antibiotics in view of the increasing antibiotic resistance^57^.

There are several limitations of this study. First, compared to the current bacteria sequence databases, the limited bacteriophage sequences in the public databases hamper the identification of bacterial host for phages and significantly limit the gut virome study. Second, whether various eradication therapies work differently on the gut virome need to be validated in future large-scale studies due to the small sample size of this pilot study. Third, a previous study revealed a time-dependent manner of the impacts of antibiotics on bacteriophages that targeted *Pseudomonas aeruginosa*^37^. In this study, we observed a decrease of the virome community 6-month after the eradication therapy and a longer observation period will be needed to fully understand the alteration of gut virome. Fourth, we relied on the similarity of predicted ORFs to Viral RefSeq to obtain viral taxonomy information, which could limit the scope of viral diversity being detected. To address this limitation, we have considered other viral database such as Gut Phage Database (GPD)^26^ which contains over 140,000 viral contigs detected from public metagenomic data. However, these viral genomes only provide taxonomy annotation at the family level. Considering Viral RefSeq has been widely used in many recent studies^20,29^, we believe this is the most appropriate approach for our study. Finally, as current metagenomic sequencing approach ignored RNA viruses and was not capable of differentiating between active and dormant phages without wet lab approaches, the role of gut eukaryotic viruses could be undervalued^58,59^. Future works targeting eukaryotic viruses and perfecting the phage database will be necessary.

In conclusion, we have demonstrated the impacts of *H. pylori* eradication in reshaping gut virome, gut microbiota, and viral-bacteria cross-kingdom interactions using metagenomic methods. Our findings highlight the changes in the previously less explored part of the gut microenvironment related to antibiotics treatment for *H. pylori*.

## Methods

### Patient characteristics, stool sample collection, and sequencing

This study was approved by the Institutional Review Board of the University of Hong Kong/Hospital Authority Hong Kong West Cluster (UW 18-413). Each patient provided written informed consent for receiving *H. pylori* eradication treatment by their attending clinicians and providing stool samples for this study. We included 44 adult patients with confirmed *H. pylori* infection, by urea breath test (C^13^-UBT) or endoscopic-based tests. For treatment naive patients (*n* = 21), clarithromycin-based triple therapy (proton pump inhibitor, amoxicillin and clarithromycin) was given. For patients who had failed previous eradication therapy (*n* = 23), levofloxacin-based quadruple therapy, bismuth quadruple therapy, or other combinations were given depending on their previous treatment regimens. The average number of prior therapies in the retreatment group was 1.91. Urea breath test (C^13^-UBT) was used to confirm the success of *H. pylori* eradication after treatment, which was the standard of care in our hospital. The authors were not involved in the treatments, all the treatment was standard of care and the detailed therapy information was provided in Supplementary Data 1. For patients who failed prior therapy, prior eradication regimens were also included in Supplementary Data 2.

Stool samples were collected at baseline, 6-week, and 6-month after the completion of the eradication therapy. Stool samples were collected using OMNIgene⋅Gut collection tubes (DNA Genotek, Ottawa, Canada) and were firstly stored at −20 °C before being transferred for DNA extraction. Whole-genome DNA was extracted according to the instruction of the QIAsymphony DSP Virus/Pathogen Kit (Qiagen, CA, USA). Briefly, 200 μl stool sample was obtained from the OMNIgene GUT collection tube and then diluted with 800 μl saline, 50 μl lysozyme (20 mg/ml), 2.5 μl mutanolysin (25 U/μl), 3 μl lysostaphin (4 U/μl) at 37 °C for one hour. Subsequently, the samples were placed in the TissueLyser II (Qiagen, cat. No. 85300) for 10 min at 30 Hz for efficient disruption of the samples and 40 μl proteinase K was applied later at 56 °C for two hours. Total DNA was then extracted from 400 μl mixture using the QIAsymphony machine with the DSP Virus/Pathogen Kit. KAPA Hyper Prep Kit (KR0961-V1.14) protocol was used for stool DNA library preparation and construction. 50 ng of extracted DNA was used for the library preparation in each sample. Briefly, the dsDNA was fragmented as end-repaired and A-tailing at 3′ end; the adaptor was ligated with an IDT dual-indexed UMI adaptor system at the terminal ends. The amplified DNA library was validated by Qubit and qPCR for quality control. After diluted to optimal concentration, Illumina NovaSeq 6000 System (Illumina, USA, Paired-end 2 × 150 bp) was used for metagenomic sequencing. The sequencing generated around 54 million reads for each sample (range from 43.35 to 68.69 million).

### Putative viral contigs identification

Raw reads were cleaned by quality control (FastQC v0.11.9), followed by removing low-quality reads and filtering human genome contamination by BBMap v38.86 (parameters minid = 0.95 maxindel = 3 bwr = 0.16 bw = 12 minhits = 2 trimq = 10)^60^. MEGAHIT v1.2.1^61^ was used to assemble the paired-end reads into contigs using the default parameter (contigs shorter than 200 bp were discarded). In total, 15,516,991 contigs were obtained from 121 samples. To identify viral contigs from assembled metagenome contigs, VirSorter v1.0.5^62^ and DeepVirFinder^63^ were used to discriminate contigs with a viral or bacterial origin. Reference database of VirSorter was constructed from Refseq viral proteins and viral genomes from published metagenomic samples. DeepVirFinder used a machine learning method for the identification of unknown and short viral sequences with high performance. For VirSorter, contigs longer than 5 kb or circular contigs over 1.5 kb were regarded as potential viral contigs. For DeepVirFinder, contigs longer than 500 were collected for the viral contig identification following a previous study^64^. A contig would be included as a candidate viral contig if it was either in VirSorter result or DeepVirFinder result.

We adopted the false positive removal procedures that were applied in construction of GVD database^13^. The protein sequences of ORFs (using Prodigal^65^) were aligned to bacterial and viral database for the verification of candidate viral contigs. Firstly, CAT^66^ used the bacteria taxonomy database constructed from NCBI non-redundant proteins and assigned a contig to bacteria or viral species by protein alignment results of ORFs. For each contig, the proportion of ORFs that can align to bacteria genes was counted. The larger the proportion was, the less likely the contig was a viral genome. If a candidate viral contig was assigned to a bacteria species and over 40% of its ORFs supported the assignment, the contig was discarded. After this step, the remaining contigs were aligned to VPF^67^, a viral gene HMM database. If a contig had at least three hits (e-value <0.05) on VPF database, the contig was a valid viral contig. Otherwise, it would align to BUSCO^68^ using hmmsearch^69^, which contained bacteria gene database as the final filtering step. Similar with CAT alignment, we calculated the ratio of ORFs that can align to BUSCO database for each contig. If the BUSCO ratio was over 0.067, the contig was pruned. The BUSCO database is composed of Hidden Markov Models (HMMs) constructed from genes that are conserved in bacteria. The database provides a score cut-off for each HMM. Genes of viral contigs were predicted by Prodigal. We applied hmmsearch to identify genes from viral contigs against HMMs in the BUSCO database. If a gene located at [x, y] in a viral contig was mapped to a BUSCO HMM with a score exceeding the database-determined cut-off, this gene was regarded as a bacteria gene. The regions excluding [x, y] are defined as non-bacteria regions. These steps resulted in 55,642 candidate contigs.

### Taxonomic annotation of viral contigs

Viral contigs in each sample were dereplicated by CD-HIT with 95% nucleotide identity over 85% alignment fraction. Specifically, for each sample, contigs were grouped into clusters from the default output of CD-HIT, and the longest contig per cluster was taken as representative. Then, the taxonomy assignment of viral contigs was performed using BLAST v2.13.0^70^ against NCBI Viral RefSeq proteins. ORFs were predicted and extracted from the assembled contigs using Prodigal. The amino acid sequences of these ORFs were further mapped to the NCBI RefSeq viral proteins. For downstream analysis, only alignment hits of over 50% alignment to viral protein sequences in the RefSeq Viral database (September 2022) in terms of sequence identity/total sequence length were considered as valid hits by BLASTP^70^ when run with default parameters (listed in Supplementary Methods). The decontam (R) package^71^ was run via frequency mode after dereplication on the assumption that contaminating DNA was present in uniform concentrations across samples and we have no negative-control samples. Viral taxonomic levels (i.e., species, genus, family, order) were predicted by a majority approach. More specifically, if over 50% of ORFs from a contig were mapped to the reference viral proteins of the same taxonomy (i.e., species, genus, family, order), the contig was assigned to this taxonomy. For example, if a contig had 10 ORFs and at least 5 of them aligned to reference proteins of the same viral species, the contig was assigned to this species. If two viral species report equal number of ORFs, the sum of alignment bit scores would resolve the ambiguity. Also, we required a contig that had at least one ORF with an alignment length of over 20 amino acids to be considered for taxonomic annotation. In the following context, viral species referred to those contigs with assigned taxonomy at species level.

In summary, we obtained 55,642 qualified viral contigs from all samples and 20,264 with a known viral taxonomy. Moreover, as revealed by a recent study that some bacterial contigs can be mis-characterized as nucleocytoplasmic large DNA viruses (NCLDV), we verified the taxonomy assignment of 2259 viral contigs that were previously mapped to NCLDV using ViralRecall (VR)^72^ by two steps. In Step 1, all qualified contigs (*N* = 2259) were searched against two viral HMM databases: GVODs (specific to NCLDV) and VOGDB (broad characterization of viral signatures) for the identification of NCLDV-specific viral orthologous (mean VR prediction score > 1). The verification confirmed the assignment of 588 NCLDV species (mean VR prediction score > 1). In Step 2, we evaluated the predicted proteins among these contigs (*N* = 588) by using the Pfam database (v.32) for the identification of virus-unique proteins (e.g., capsid protein) or cellular organisms conserved proteins (e.g., heat-shock proteins). Further evaluation from predicted proteins of these NCLDV contigs suggested none of them has virus unique protein. Thus all 2259 previously assigned NCLDV contigs were excluded from downstream analysis. Finally, 18,005 valid viral contigs with known taxonomy (International Committee on Taxonomy of Viruses, ICTV taxonomy release #37, 2021) were used for downstream analysis. At contigs level, the richness of contigs decreased after the eradication with similar trend as at the species level. Furthermore, viral species assigned to RNA viruses (N = 1533) were removed since our study did not consider the viral RNA during extraction. The dereplicated contig abundance was calculated as the RPKM per known viral species (normalized by contigs length and sequence depth in each sample). The abundance of each taxonomy level was calculated as the sum of the contigs shared the same taxonomic identity (i.e., the abundance of *Caudoviricetes* class was the RPKM sum of *Caudoviricetes* contigs). Counts smaller than 0.5 were removed to reduce noise. Finally, we generated a taxonomy table with 3581 viral species. The virome diversity (alpha diversity indices and beta diversity) was based on the abundance of virome species-level using the *vegan* package in R. The core, common, and unique viral species were defined according to the species prevalence following the below criteria: (1) core species: viral species found in >50% of the samples; (2) common: viral species found 20–50% of the samples; (3) unique species: viral species found in <20% of all the samples.

### Microbiota taxonomic profiling

The taxonomy assignment for bacteria genomes was performed on assembled contigs using *k-mer*-based Kraken2 against the NCBI Reference Sequence Database (RefSeq). Kraken2 was an ultrafast and highly accurate tool for metagenomic sequences classification. The abundance of each taxonomy level (species, genus, family, and phylum) was calculated and further normalized as RPKM (reads per kilobase per million reads). The microbial alpha diversity indices (species richness, Shannon index, and Simpson index) were calculated based on the relative abundance of bacteria species for each sample using the *vegan* package in R. For the microbial community differentiation (beta-diversity) between three different time points, the unweighted Bray-Curtis distance, was calculated using the *phyloseq* package in R.

The workflow of the sequencing data processing, viral contigs identification, and viral taxonomy annotation was presented in Supplementary Fig. 12.

### Antibiotic resistance gene profiling

Assembled contigs were mapped to the Comprehensive Antibiotic Resistance Database (CARD)^73^ using the Resistance Gene Identifier (RGI) v5.11 with default parameters as described previously^31^. The abundance of the ARGs was calculated using ShortBRED (95% identity and 95% length coverage)^74^. The RPKM abundance of ARGs was calculated.

### Statistical analysis

All statistical analysis and figures were performed by the R software (v4.0.3) unless otherwise stated. For microbiome diversity, the two-sided Wilcoxon signed-rank test or Manny–Whitney U test was used for paired and unpaired samples. Benjamini–Hochberg (FDR) was applied for multiple comparison correction. MaAsLin2 package in R was used to identify differential abundance on microbiome taxonomy level with linear model (original *p* value <0.05 with *q* values <0.05 were considered significant; formula adjusted for age, gender, treatment regimens, drug durations, raw reads with a baseline set as the reference group)^75^. Principal coordinates analysis (PCoA) of beta-diversity of gut microbiota between or within treatment groups was visualized based on chi-square distance matrices. Permutational multivariate analysis of variance (PERMANOVA) was then performed to compare microbiota community dissimilarity using analysis of variance using distance matrices (ADONIS) for 999 permutations. For the comparisons of the average Bray-Curtis dissimilarity distance, permutation tests (99,999 times) were used. Spearman’s rank correlation was used to evaluate the association between bacteriophage-bacteria taxa, Benjamini–Hochberg correction with cut-off value 0.05 applied for all paired correlations. Pearson correlation was applied to evaluate the alpha diversity correlation between viral species and bacteria species.

### Reporting summary

Further information on research design is available in the Nature Portfolio Reporting Summary linked to this article.

## Supplementary information

Supplementary information

Description of Additional Supplementary Files

Dataset 1 & 2

Supplementary Data 3

Reporting Summary

## Data Availability

The dataset supporting this article is available in the NCBI Sequencing Read Archive under BioProject ID: PRJNA749138. Other public databases used included CARD (https://card.mcmaster.ca/), BUSCO (https://busco.ezlab.org/), and NCBI virus/bacteria/refseq (https://www.ncbi.nlm.nih.gov/refseq/).

## Code availability

Scripts used for the bioinformatic analysis can be found at: https://github.com/hbyaoherbert/ARG_Virus.
