# Supplementary material for: Altered human gut virome in patients undergoing antibiotics therapy for Helicobacter pylori
Source: Nat Commun. 2023 Apr 17;14:2196. doi: 10.1038/s41467-023-37975-y (PMC10110541; doi:10.1038/s41467-023-37975-y)
Supplement: Supplementary file 1 — Supplementary information [file 41467_2023_37975_MOESM1_ESM.pdf]

Supplementary information

Methods

BlastP parameters:

- Max target sequence =100
- Short queries = True
- Expect threshold = 0.05
- Word size = 6
- Max matches in a query range = 0
- Matrix = BLOSUM62
- Gap costs = existence: 11, extension: 1
- Compositional adjustments: conditional compositional score matrix adjustment
- Filtering low complexity regions = False
- Mask for lookup table only = False
- Mask lower case letter = False

Supplementary Figures

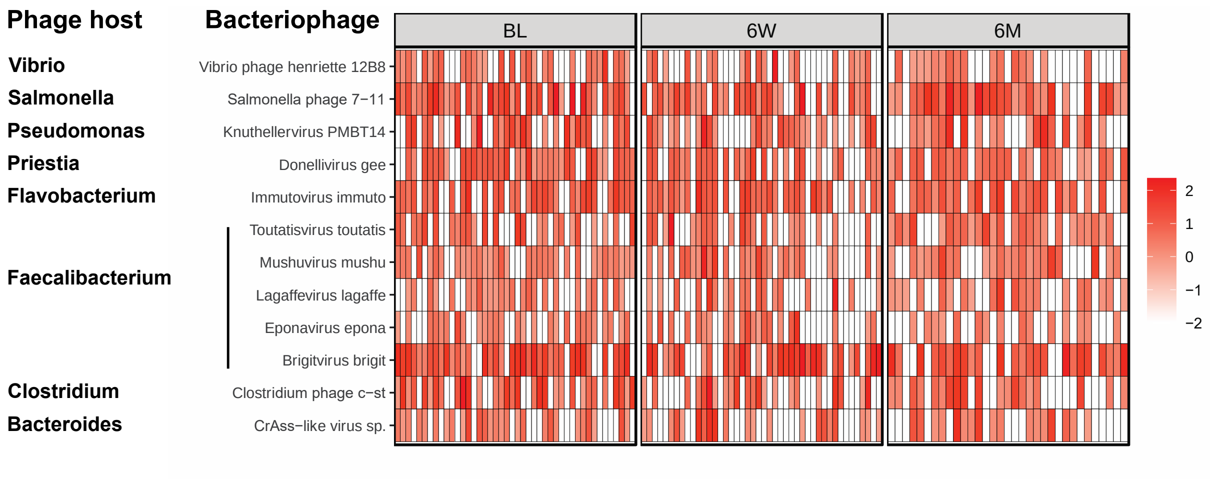

Supplementary Fig. 1: Presence and abundance of core viruses

Heatmap showing the presence and abundance of 12 core bacteriophages at baseline (BL), 6-week (6W) and 6-month (6M). White cells indicated the absence of the viral species while red cells indicated the presence of the viral species. Color scale presented the Log_{10} (RPKM) abundance of the viral species. The theoretical host of the bacteriophages were added.

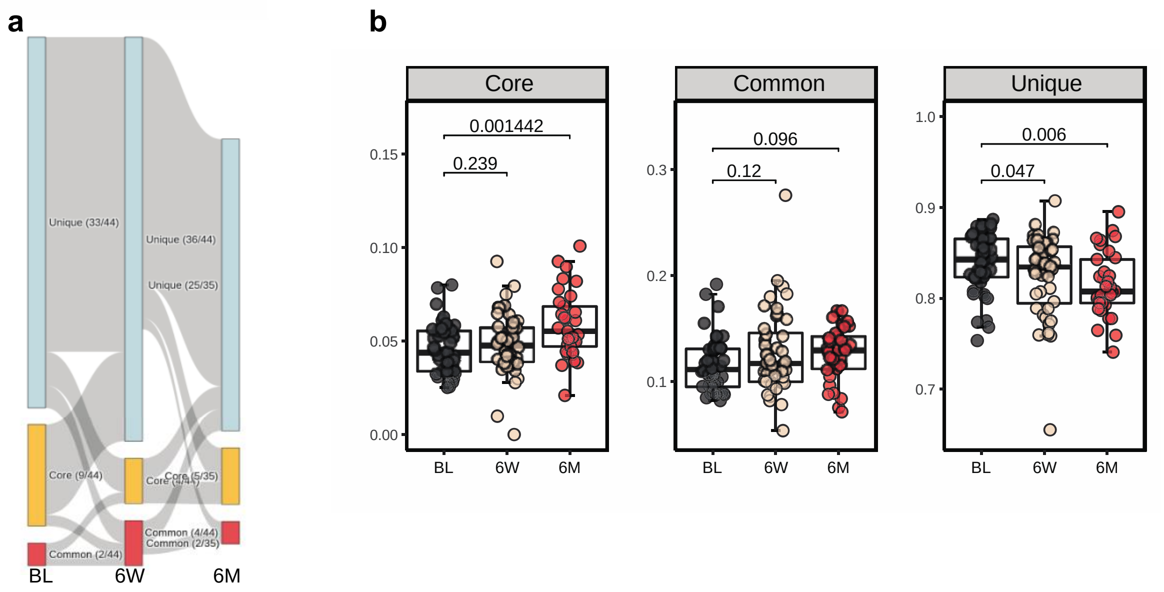

Supplementary Fig. 2: The richness and abundance of viruses at each prevalence level

(a) Sankey diagram showing the transition of the virus categories (core/common/unique) at three time point in each sample. The Sankey diagram the movement of the maximum virus in each subject. (b) Boxplot showing the significantly altered composition of core, common and unique viruses after the eradication therapy at 6-week (N=44) and 6-month (N=33) compared to baseline (N=44) (two-sided Wilcoxon test followed by Benjamini-Hochberg FDR method for multiple comparisons correction). The boxes denote the lower 25% quantile, upper 75% quantile, and center line the median, with whiskers extending to a limit of ± 1.5 interquartile ranges (IQRs).

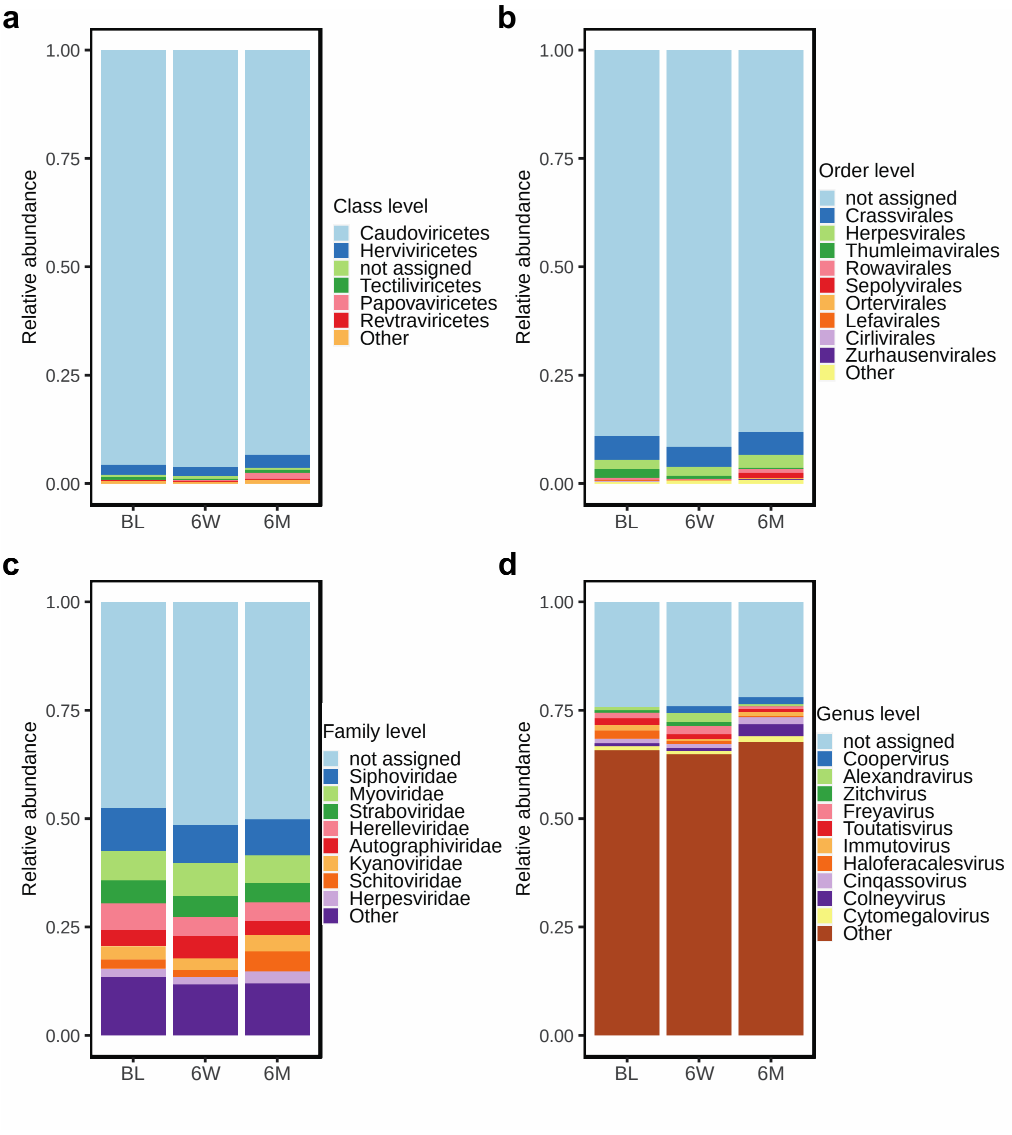

Supplementary Fig. 3: Composition of gut virome composition at different taxonomy level. (a-d) Stacked plot showing the virome composition at (a) Class, (b) Order, (c) Family, and (d) Genus level. *Caudoviricetes* class was dominant while most of the viral taxonomy were not assigned at family or genus level according to the latest ICTV taxonomy release (#37).

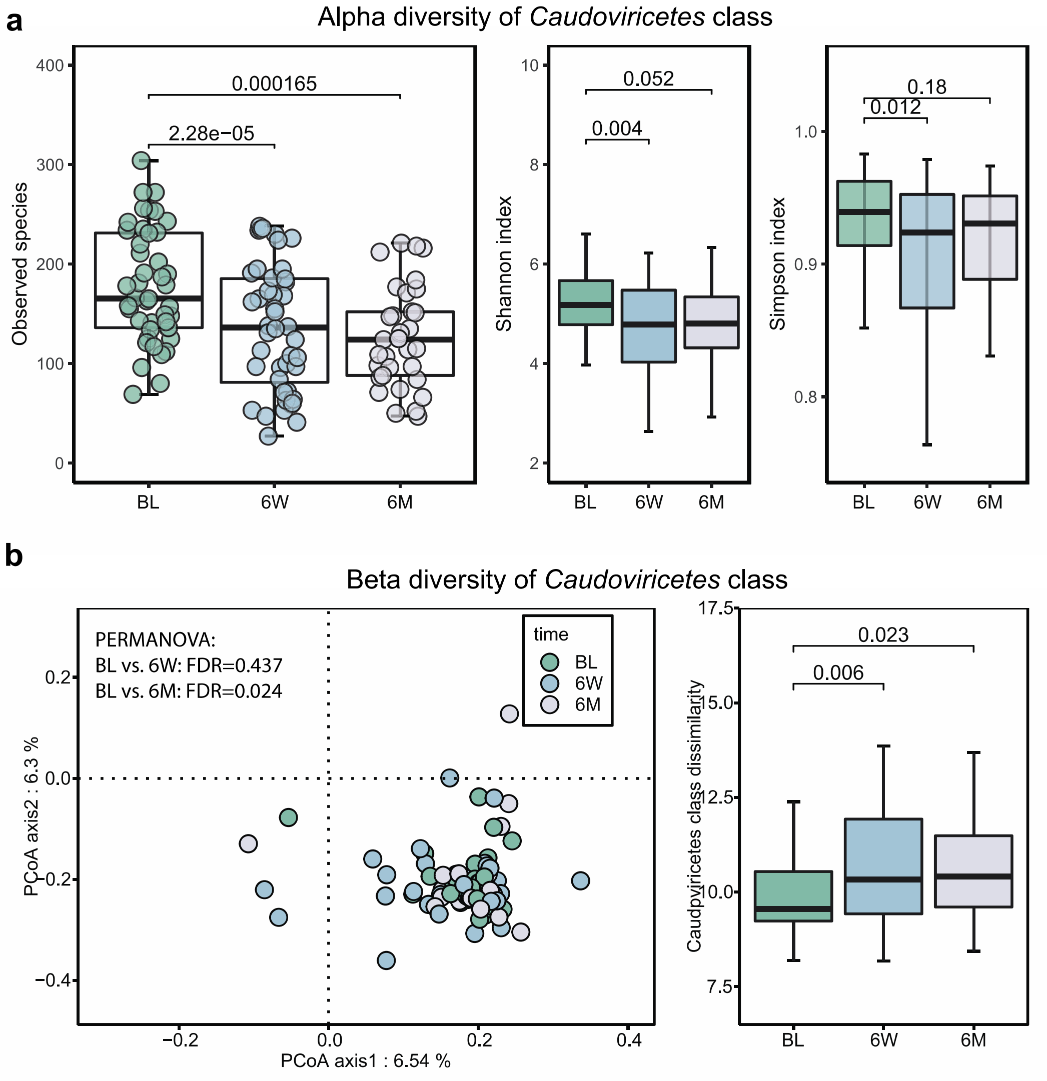

Supplementary Fig. 4: Alteration in biodiversity of the *Caudoviricetes* class.

(a) Significantly decreased alpha diversity indices of the *Caudoviricetes* class in richness (left), Shannon index (medium), and Simpson index (right) after the eradication therapy at either 6-week (N=44) or 6-month (N=33) compared with baseline (N=44). Alpha diversity indices were tested using Wilcoxon tests (two-sided) and adjusted by BH method for multiple comparisons. (b) PCoA plot showing the significantly separated *Caudoviricetes* class community after the treatment (left) and increased *Caudoviricetes* class dissimilarity (right) at 6-week (N=44) and 6-month (N=33) as compared to baseline (N=44).

PERMANOVA test was used for the beta diversity and adjusted by BH method. The boxes denote the lower 25% quantile, upper 75% quantile, and center line the median, with whiskers extending to a limit of ± 1.5 interquartile ranges (IQRs).

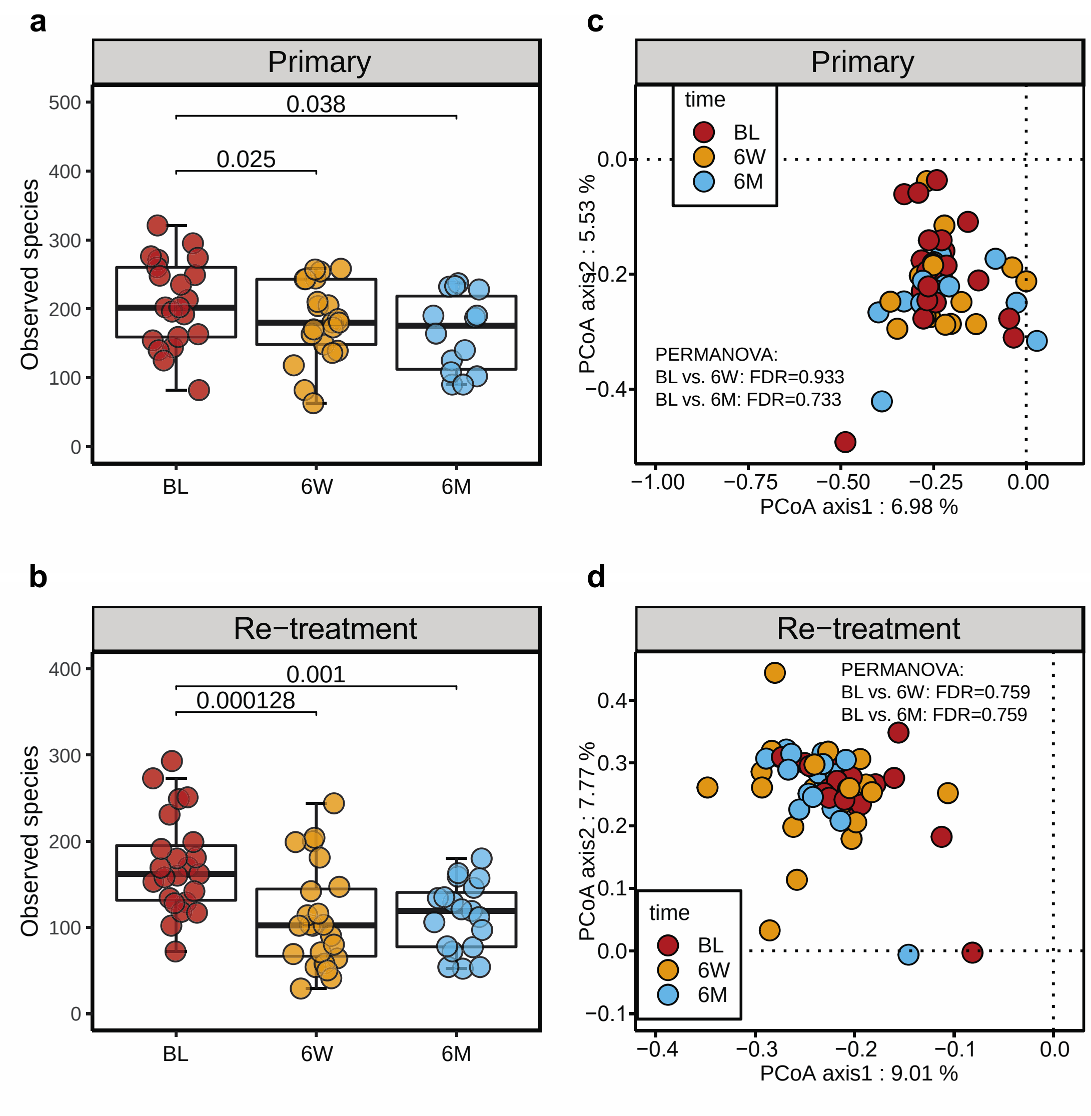

Supplementary Fig. 5: Both primary and retreatment lead to similar alteration of the gut virome.

(a-b) Significantly altered alpha diversity (richness) after the eradication therapy in (a) primary and (b) retreatment group. Alpha diversity was tested using two-sided Wilcoxon tests and adjusted by BH method. (c-d) PCoA plots showing the viral community difference

after (c) primary and (d) retreatment. Beta diversity was tested using PERMANOVA tests. In the primary group, there were 21 patients at baseline, 21 at 6-week, and 14 at 6-month; in the re-treatment group, there were 23 patients at baseline, 23 at 6-week, and 19 at 6-month. The boxes denote the lower 25% quantile, upper 75% quantile, and center line the median, with whiskers extending to a limit of ± 1.5 interquartile ranges (IQRs).

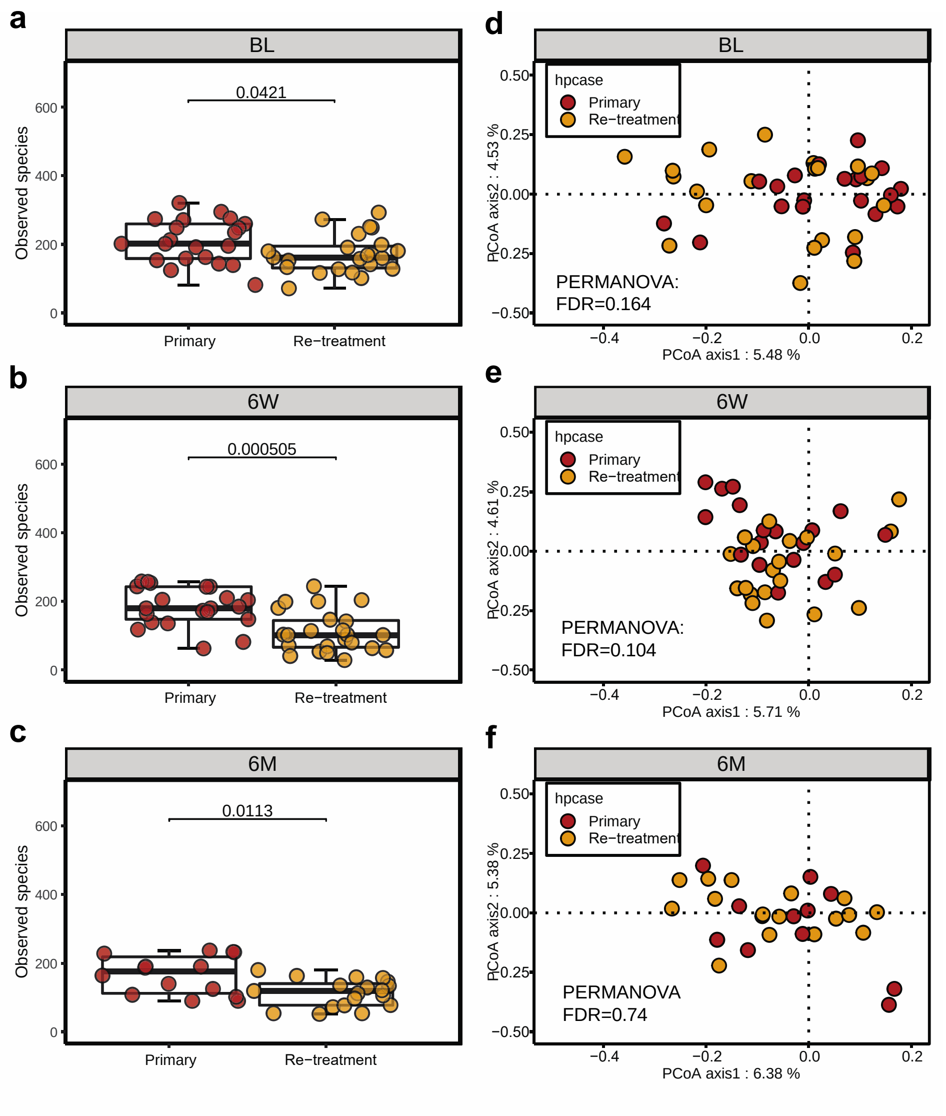

Supplementary Fig. 6: Difference of gut virome diversity and community between primary and retreatment groups at each time point.

(a-c) Lower virus richness in the retreatment group as compared to the primary group at (a) baseline (primary, N=21; re-treatment, N=23), (b) 6-week (primary, N=21; re-treatment, N=23), and (c) 6-month (primary, N=14; re-treatment, N=19). Two-sided Wilcoxon test was used for the comparison between groups. (d-f) PCoA plots suggested no significant viral structure difference between primary and retreatment group at (d) baseline, (e) 6-week, and (f) 6-month. PERMANOVA test was used to determine the beta diversity difference. The boxes denote the lower 25% quantile, upper 75% quantile, and center line the median, with whiskers extending to a limit of ± 1.5 interquartile ranges (IQRs).

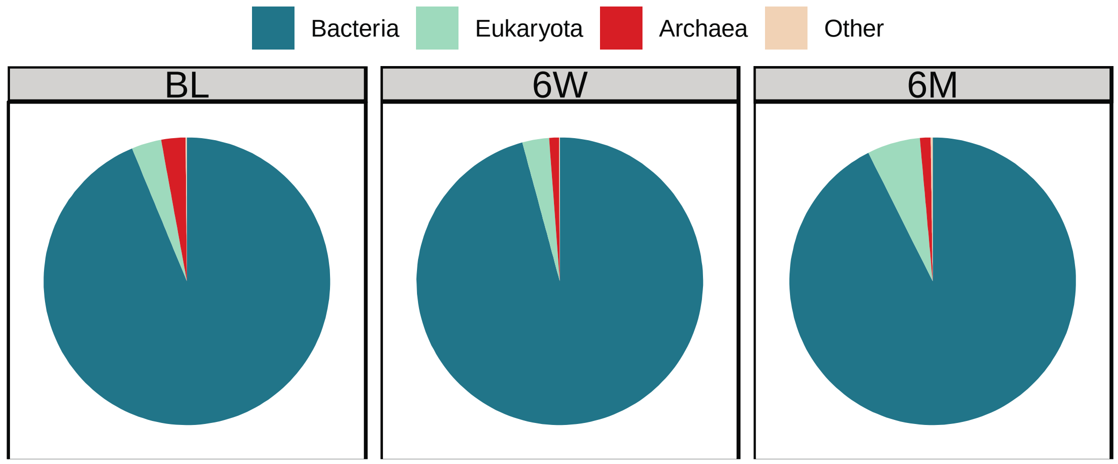

Supplementary Fig. 7: The composition of bacteriophages, eukaryotic viruses, archaea, and other viruses calculated at the species level.

Pie chart showing the abundance composition of viruses classified according to the theoretical hosts. Other includes hosts from plants, marine, environmental, unclassified etc.

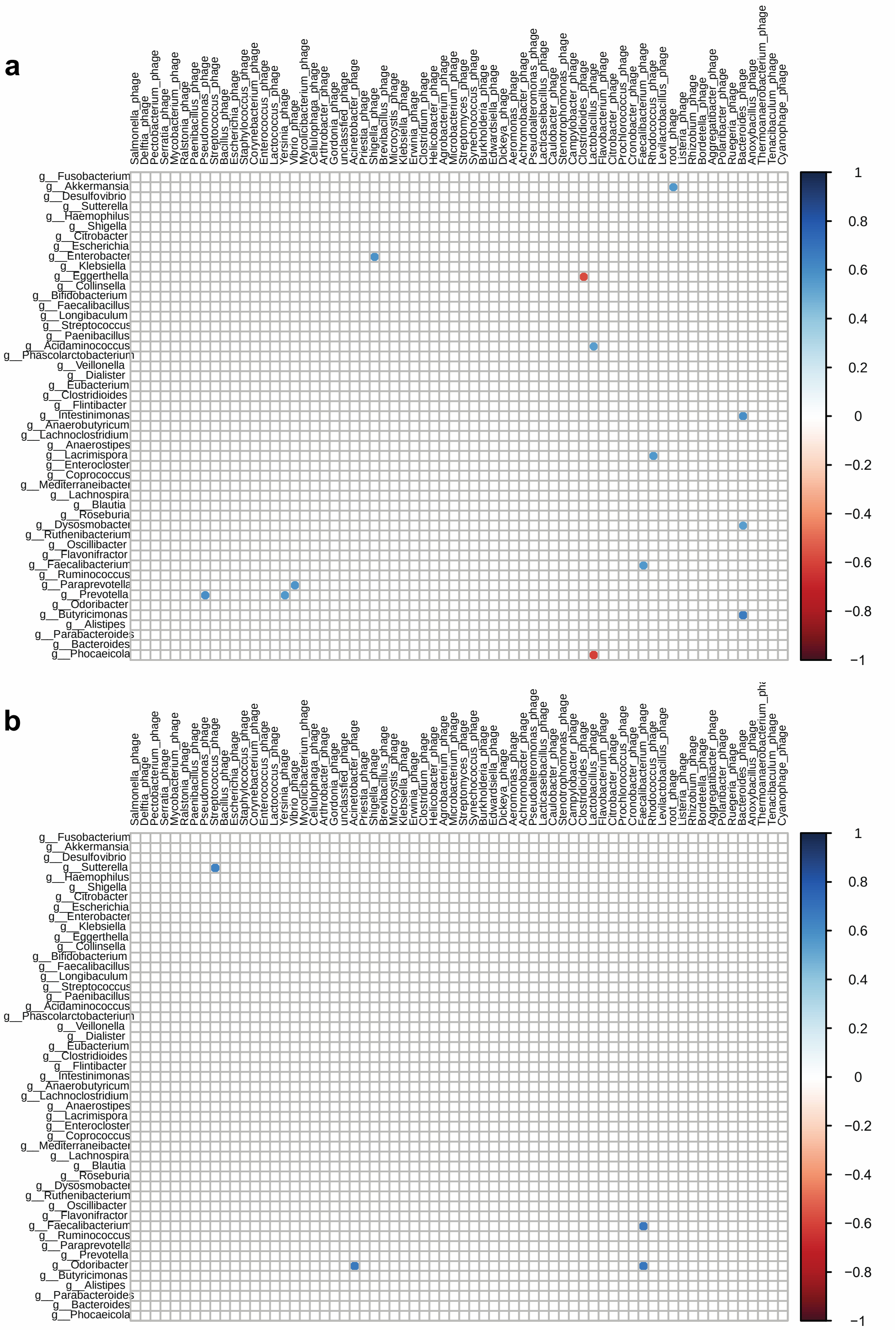

Supplementary Fig. 8: Phage-bacteria correlation pattern at baseline and 6-month after the eradication therapy.

(a-b) Bacteriophage-bacteria correlations at (a) baseline and (b) 6-month. Only significant correlations (BH method for multiple comparisons correction, adjusted p < 0.05) were displayed. Detailed p-values were included in the Supplementary Data 3.

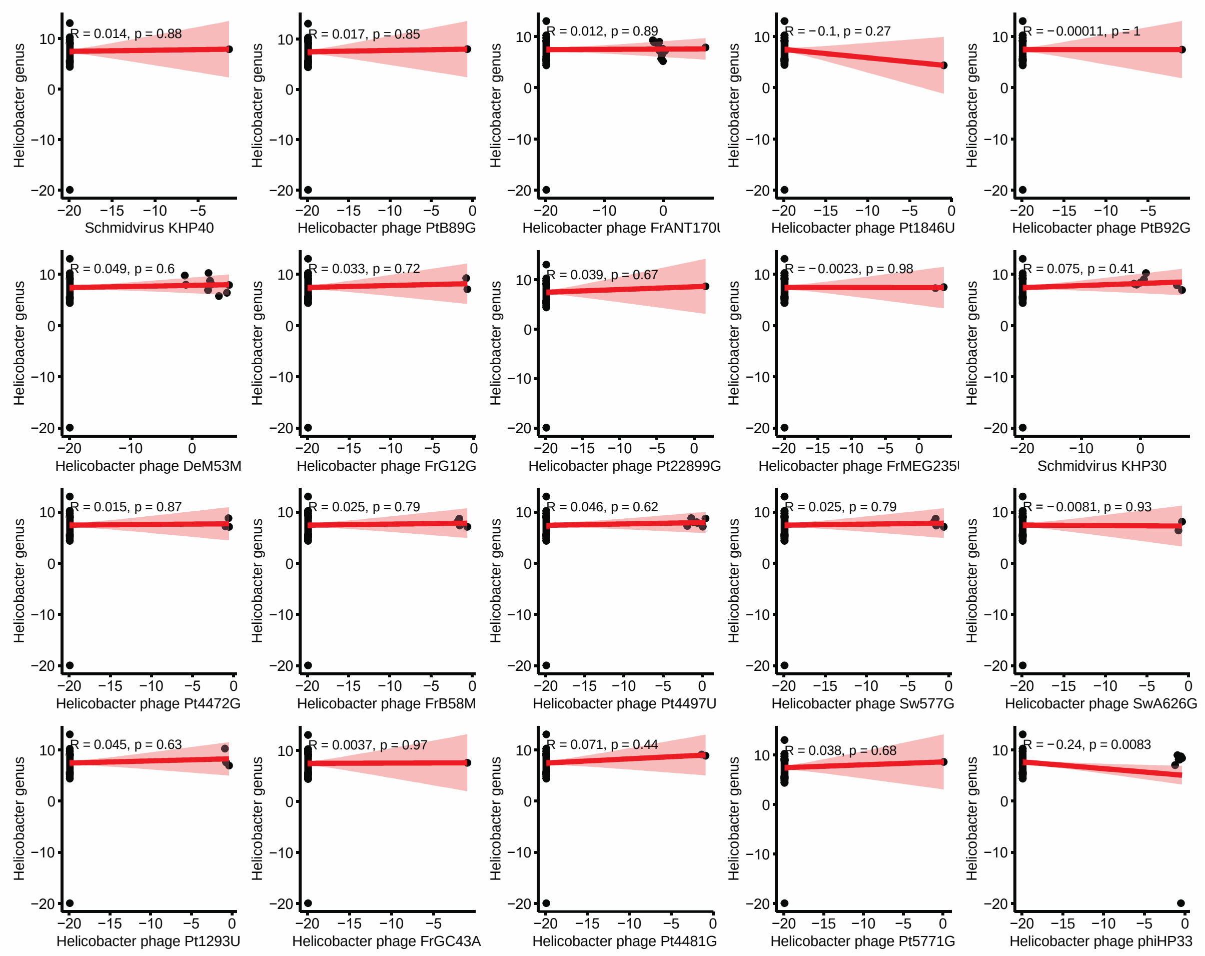

Supplementary Fig. 9: Association between *Helicobacter* genus and *Heliobacter* phages.

Pearson correlation (two-sided) between the abundance of *Helicobacter* genus and *Helicobacter phages*. *Helicobacter* phage was the sum of all the *Helicobacter* phages.

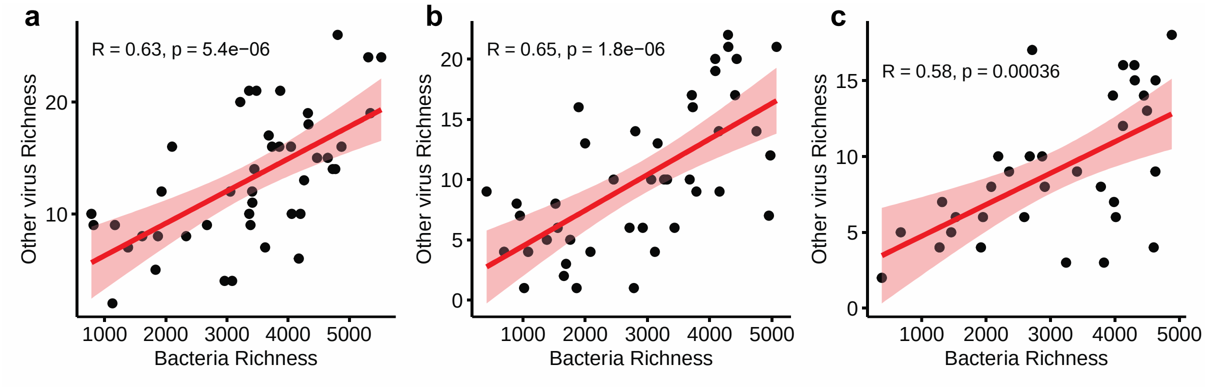

Supplementary Fig. 10: Altered correlations between the richness of bacteria and other viruses after the eradication therapy.

Pearson correlation (two-sided) analysis of alpha diversity index indicated strong and positive correlations at (a) baseline, (b) 6-week, and (c) 6-month between bacteria and other viruses (non-bacteriophage, including eukaryotic viruses, environmental viruses, and those unclassified viruses).

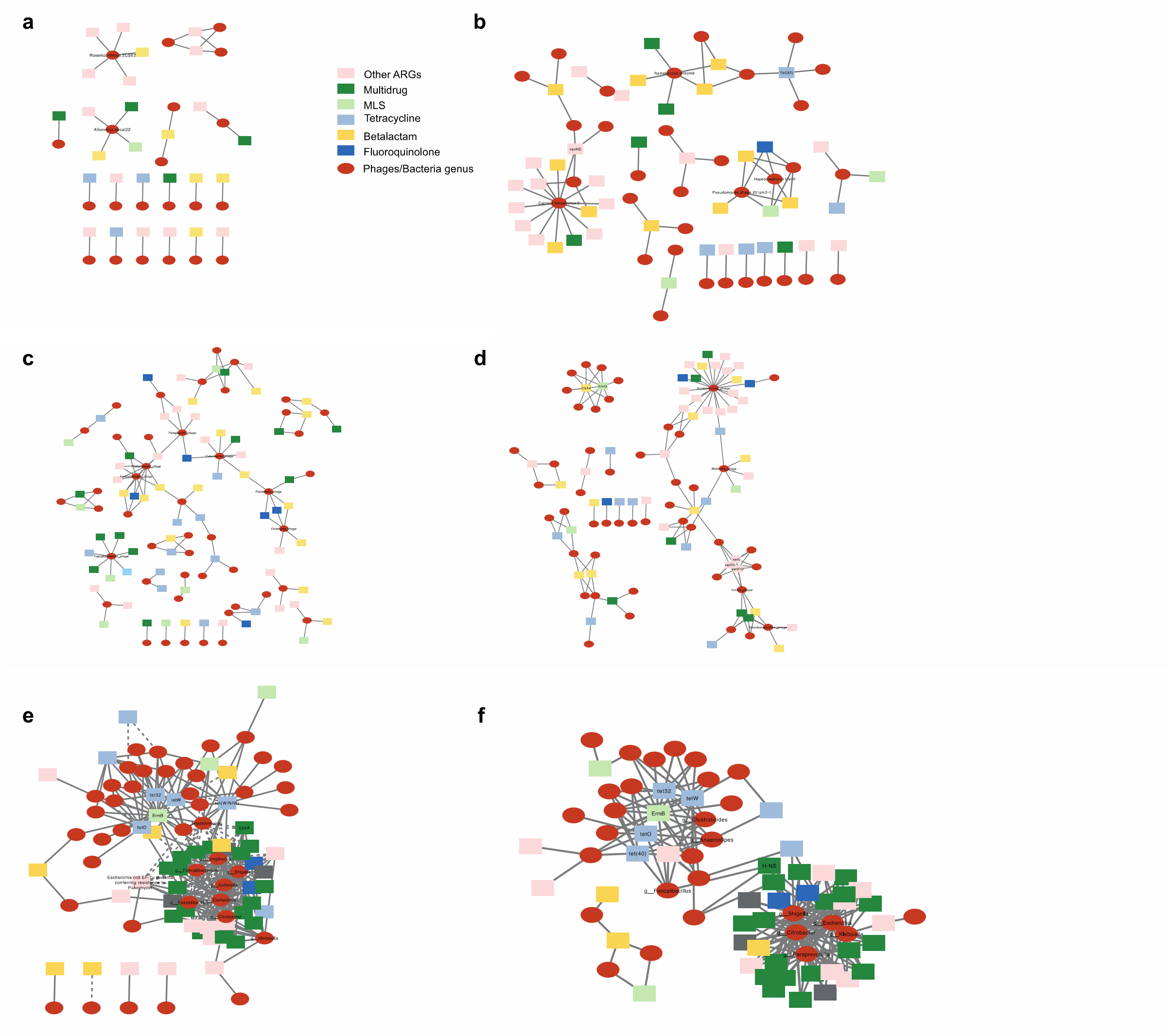

Supplementary Fig. 11: Network analysis between bacteriophages and antibiotic resistance genes (ARGs) after the eradication therapy.

(a-b) Network analysis showing the spearman correlations between single bacteriophage and unique ARGs at (a) 6-week and (b) 6-month after treatment. (c-d) Network analysis showing the correlations between summarized phages (bacteriophages share the same bacteria genus) and unique ARGs. Circles represent the bacteriophages/bacteria genus and squares represented the unique antibiotic genes. The color of the circles indicated the class of ARGs. Purple lines indicated the positive correlations and grey lines indicated the negative correlations. Only the correlations with adjusted (BH method for multiple comparisons corretion) p-value <0.05 were included in the network. Cytoscape v3.9.0 was

used for the visualization of the network[5]. Detailed p-values were included in Supplementary Data 3.

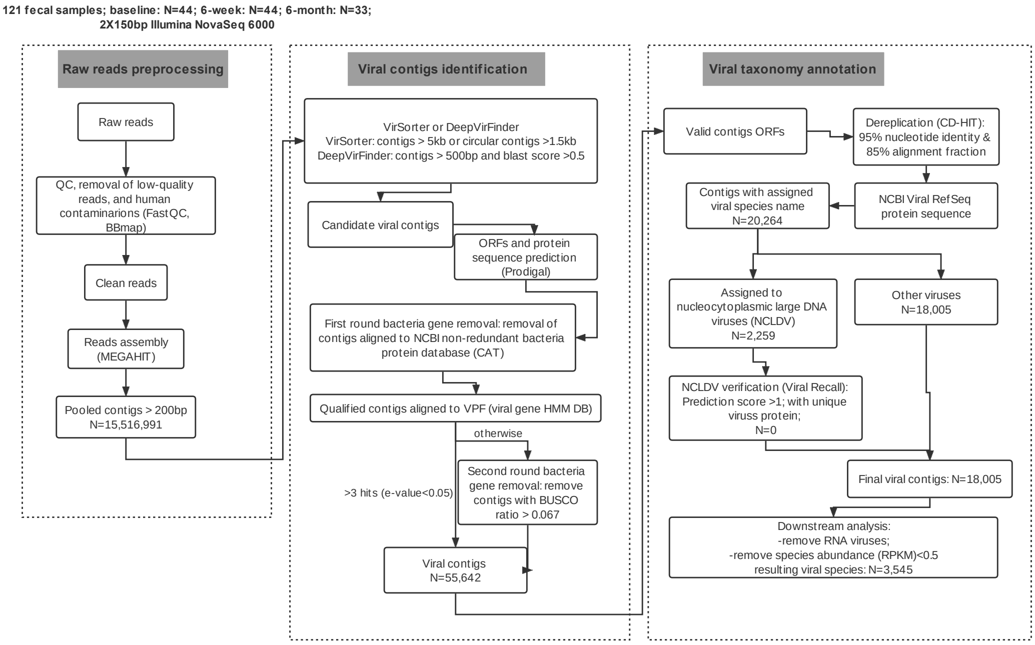

Supplementary Fig. 12. The workflow of the raw data processing, viral contigs identification, and viral taxonomy annotation.

The main workflow of this study was composed of three parts: raw data preprocessing, viral contigs identification, and viral taxonomy annotation. Viral contigs identification involves viral contigs identification and 2 rounds bacterial genome removal. The viral taxonomy annotation also includes taxonomy annotation and NCLDV contigs verification.
