# Supplementary material for: Altered human gut virome in patients undergoing antibiotics therapy for Helicobacter pylori
Source: Nat Commun. 2023 Apr 17;14:2196. doi: 10.1038/s41467-023-37975-y (PMC10110541; doi:10.1038/s41467-023-37975-y)
Supplement: Supplementary file 2 — Description of Additional Supplementary Files [file 41467_2023_37975_MOESM2_ESM.pdf]

Description of Additional Supplementary Files

File Name: Supplementary Data 1
Description: **Current HP eradication regimens for all patients**

File Name: Supplementary Data 2
Description: **Prior failed HP eradication regimens for patients who failed prior current treatment**

File Name: Supplementary Data 3
Description: **MaAslin2 output for the microbiome taxonomy & microbiome correlation analysis**
