# Supplementary material for: Altered human gut virome in patients undergoing antibiotics therapy for Helicobacter pylori
Source: Nat Commun. 2023 Apr 17;14:2196. doi: 10.1038/s41467-023-37975-y (PMC10110541; doi:10.1038/s41467-023-37975-y)
Supplement: Supplementary file 5 — Reporting Summary [file 41467_2023_37975_MOESM5_ESM.pdf]

nature portfolio

Corresponding author(s): Wai K Leung
Last updated by author(s): Apr 3, 2023

Reporting Summary

Nature Portfolio wishes to improve the reproducibility of the work that we publish. This form provides structure for consistency and transparency in reporting. For further information on Nature Portfolio policies, see our Editorial Policies and the Editorial Policy Checklist.

Statistics

For all statistical analyses, confirm that the following items are present in the figure legend, table legend, main text, or Methods section.

n/a	Confirmed	
☐	☒	The exact sample size (n) for each experimental group/condition, given as a discrete number and unit of measurement
☐	☒	A statement on whether measurements were taken from distinct samples or whether the same sample was measured repeatedly
☐	☒	The statistical test(s) used AND whether they are one- or two-sided<br>*Only common tests should be described solely by name; describe more complex techniques in the Methods section.*
☐	☒	A description of all covariates tested
☐	☒	A description of any assumptions or corrections, such as tests of normality and adjustment for multiple comparisons
☐	☒	A full description of the statistical parameters including central tendency (e.g. means) or other basic estimates (e.g. regression coefficient) AND variation (e.g. standard deviation) or associated estimates of uncertainty (e.g. confidence intervals)
☐	☒	For null hypothesis testing, the test statistic (e.g. F, t, r) with confidence intervals, effect sizes, degrees of freedom and P value noted<br>*Give P values as exact values whenever suitable.*
☒	☐	For Bayesian analysis, information on the choice of priors and Markov chain Monte Carlo settings
☒	☐	For hierarchical and complex designs, identification of the appropriate level for tests and full reporting of outcomes
☐	☒	Estimates of effect sizes (e.g. Cohen's d, Pearson's r), indicating how they were calculated

Our web collection on statistics for biologists contains articles on many of the points above.

Software and code

Policy information about availability of computer code

Data collection	NCBI SRA: PRJNA749138;
Data analysis	FastQC v0.11.9; BBMap v38.86; MEGAHIT v1.2.1; VirSorter v1.0.5; DeepVirFinder; Prodigal; CD-HIT; BLAST v2.13.0; ViralRecall; ShortBRED; R software v4.0.3; MaAslin2 R package;<br>Scripts used for the bioinformatic analysis can be found in: https://github.com/hbyaoherbert/ARG_Virus.

For manuscripts utilizing custom algorithms or software that are central to the research but not yet described in published literature, software must be made available to editors and reviewers. We strongly encourage code deposition in a community repository (e.g. GitHub). See the Nature Portfolio guidelines for submitting code & software for further information.

Data

Policy information about availability of data

All manuscripts must include a data availability statement. This statement should provide the following information, where applicable:

- Accession codes, unique identifiers, or web links for publicly available datasets
- A description of any restrictions on data availability
- For clinical datasets or third party data, please ensure that the statement adheres to our policy

The dataset supporting the conclusions of this article is available NCBI Sequencing Read Archive under BioProject ID: PRJNA749138. Other public databases used included CARD (https://card.mcmaster.ca/), BUSCO (https://busco.ezlab.org/), and NCBI virus/ bacteria/ refseq (https://www.ncbi.nlm.nih.gov/refseq/).

Human research participants

Policy information about studies involving human research participants and Sex and Gender in Research.

Reporting on sex and gender	This study includes 18 male and 26 female. All the gender information is provided in the supplementary table.
Population characteristics	All patients are from Hong Kong, aged from 26 to 74, diagnosed with active Helicobacter pylori infection.
Recruitment	Adult patients with confirmed H. pylori infection, by urea breath test (C13-UBT) or endoscopic-based tests, from the Queen Mary Hospital of Hong Kong. Antibiotic treatment of H. pylori were regional based on the district resistance prevalence, which may resulted in the potential bias of the results of this study.
Ethics oversight	Institutional Review Board of the University of Hong Kong/Hospital Authority Hong Kong West Cluster

Note that full information on the approval of the study protocol must also be provided in the manuscript.

Field-specific reporting

Please select the one below that is the best fit for your research. If you are not sure, read the appropriate sections before making your selection.

☒ Life sciences ☐ Behavioural & social sciences ☐ Ecological, evolutionary & environmental sciences

For a reference copy of the document with all sections, see nature.com/documents/nr-reporting-summary-flat.pdf

Life sciences study design

All studies must disclose on these points even when the disclosure is negative.

Sample size	Sample size was estimated from previous studies and no statistical method had been used to predetermine the sample size. Studies focus on the metagenomic studies included around 50 subjects.
Data exclusions	No data excluded.
Replication	The whole-genome sequencing of DNA replication includes the entire genome in a single subject, thus no other replication method were applied. To lower the risk, all the multiple comparisons were corrected.
Randomization	The patients were grouped according to prior HP treatment history (Primary=treatment naive; Retreatment = at least failed one prior HP regimen).
Blinding	Blinding not relevant to this study. The randomization doesn't affect the differential treatment, the assessment of the outcomes, or biased estimation of the treatment effect in this study.

Reporting for specific materials, systems and methods

We require information from authors about some types of materials, experimental systems and methods used in many studies. Here, indicate whether each material, system or method listed is relevant to your study. If you are not sure if a list item applies to your research, read the appropriate section before selecting a response.

Materials & experimental systems

n/a	Involved in the study	
☒	☐	Antibodies
☒	☐	Eukaryotic cell lines
☒	☐	Palaeontology and archaeology
☒	☐	Animals and other organisms
☒	☐	Clinical data
☒	☐	Dual use research of concern

Methods

n/a	Involved in the study	
☒	☐	ChIP-seq
☒	☐	Flow cytometry
☒	☐	MRI-based neuroimaging
